# Discontinuation of the Clozapine Risk Evaluation and Mitigation Strategy and Implications in Movement Disorders

**DOI:** 10.1002/mdc3.70197

**Published:** 2025-06-28

**Authors:** Charles Palmer, Alexander McClanahan, Federico Rodriguez‐Porcel

**Affiliations:** ^1^ Department of Psychiatry and Behavioral Sciences Medical University of South Carolina Charleston SC USA; ^2^ Movement Disorders Division Medical University of South Carolina Department of Neurology Charleston SC USA

**Keywords:** dementia with Lewy bodies, Parkinson's disease, psychosis

## Background

When it comes to treating psychosis in Parkinson's disease (PD) and dementia with Lewy Bodies (DLB), no medication matches the efficacy and safety profile of clozapine. This statement is supported by multiple open‐label studies and two double‐blind, placebo‐controlled trials.^1^, ^2^ Yet, clozapine remains largely underutilized in PD and DLB in practice, typically considered as a last resort.^3^ To some extent this stems from the establishment of a Risk Evaluation and Mitigation Strategy (REMS) program by the US Food and Drug Administration (FDA), which served the purpose of closely monitoring for neutropenia, a rare but potentially lethal adverse effect. This increased the perception of risk as well as the follow‐up burden on the patient and clinician, limiting the use of clozapine. The recent discontinuation of the REMS program opens up an opportunity to revisit the use of clozapine in clinical practice. Here, we seek to review the evidence of clozapine, discuss the reasons for its limited use during the REMS era and its implications, and ending with a perspective of the role of clozapine in movement disorders.

## Evidence of Clozapine in the Treatment of Psychosis in PD and DLB

Clozapine remains one of the most highly effective treatments for psychosis in PD.^4^ There are two randomized, double‐blinded, placebo‐controlled trials^1^, ^2^ with similar design (60 patients across multiple sites, low dose of clozapine [starting dose of 6.25 mg, with mean doses of 25 mg^2^ vs 36 mg^1^]), both of which show significant improvement in psychotic symptoms and overall functioning with a low rate of adverse events. Open‐label and retrospective studies agree with these trials, showing a ~70% improvement rate in hallucinations and delusions. Wagner et al in 1996 found improvement of psychosis in 76% of 49 patients^5^ and Trosch et al in 1998 found symptom resolution in 54% and “marked benefit” in 36% of 172 patients.^6^ More recently, Gomide et al in 2008 showed “significant” improvement of psychosis in 20% and partial improvement in 46% of 26 patients^7^ and Hack et al in 2014 showed complete improvement in 33% and partial improvement in 33%^8^ of 36 patients. In each of these studies, clozapine dose was less than 100 mg daily (ranging 16 mg^5^–70 mg^7^) and all patients across studies had significantly progressed disease with a Hoehn and Yahr stage of 2.5–3. The most common and treatment‐limiting side effect across studies was sedation. For a comprehensive review of the data on this subject, see Table S1.

There are less data available for DLB and no randomized controlled trials. Case‐report level data shows that out of seven cases, five had improvements in psychosis, and two developed confusion and behavioral symptoms.^9^ Clinically, clozapine is used as an adjunctive agent in cases where patients remain severely symptomatic despite the use of an acetylcholinesterase inhibitor.^4^

While there is significant concern for severe neutropenia, nearly all data comes from the schizophrenia population, with estimates of this risk ranging from 0.38% to 2.0%, and a 2018 meta‐analysis reporting an incidence of 0.9% (95% CI: 0.7–1.1%).^10^ In all of the aforementioned trials for PD psychosis and DLB, the discontinuation rate due to neutropenia was limited to single cases, in occasions described as leukopenia^5,7^ but not clearly severe neutropenia and in all other trials neutropenia cases were either transient or absent.^1^, ^2^ One single‐center retrospective analysis showed an incidence of 8.3% of transient neutropenia with no cases of agranulocytosis.^8^

Comparatively, pimavanserin is tolerable and efficacious in PD and DLB,^4^, ^11^ but may have a more modest benefit, with one large randomized, double‐blind placebo‐controlled trial (*n* = 200) showing 37% improvement in PD psychosis compared to placebo.^12^ Data for DLB is limited to preliminary evidence with very small samples.^13^ Pimavanserin takes 4–6 weeks to reach efficacy^12^ compared to clozapine's rapid onset of action.^14^ Quetiapine remains the more commonly used antipsychotic in clinical practice despite a lack of efficacy data, with two randomized controlled trials for Parkinson's psychosis^15^, ^16^ and one for dementia with Parkinsonism (including DLB)^17^ failing to separate from placebo. In two large network meta‐analyses of antipsychotic usage for PD psychosis (*n* = 17 studies;^18^ *n* = 16 studies^19^), pimavanserin was found to be more efficacious than placebo^18^, ^19^ while quetiapine was found to be either “possibly efficacious”^18^ or not efficacious at all.^19^ In both analyses, clozapine was the most effective option. Of interest, one study evaluated clozapine in 27 patients who had not responded to pimavanserin and found that efficacy rates for clozapine were 41% markedly effective, 22% moderately effective, and 18% somewhat effective, with only one discontinuation (due to thrombocytopenia) and five patients with excessive sedation.^20^

Other atypical antipsychotics including olanzapine, risperidone, and aripiprazole are typically not tolerated due to worsening of Parkinsonism^21^, ^22^, ^23^ and first‐generation antipsychotics are contraindicated in this population due to significant risk for side effects.^24^

## The REMS Program: Motivation and Consequences

The risk of severe neutropenia with clozapine led the FDA to establish the Risk Evaluation and Mitigation Strategy (REMS) program, which historically obligated every patient on the medication to follow a uniform blood draw schedule or risk losing access to the drug. The program initially required weekly blood monitoring for six months, followed by biweekly monitoring for the next six months, and monthly monitoring thereafter. Providers were tasked with manually inputting each patient's absolute neutrophil count (ANC) in accordance with the established monitoring schedule. Pharmacies then had to verify compliance via the REMS database before dispensing clozapine; failure to have updated ANC records resulted in the medication being withheld.

Although introduced with the noble goal of enhancing patient safety, in practice the REMS program became excessively burdensome for both patients and healthcare providers.^25^ It placed significant demands on already time‐constrained clinicians, particularly in settings lacking sufficient administrative support, in pharmacies unfamiliar with managing the medication, or when logistical barriers delayed necessary laboratory testing. One study showed that frequent blood draws were the main reason for discontinuation of clozapine, particularly in the admission to nursing homes.^8^ Equally important, patients struggling with psychosis and their caregivers already faced disease‐related challenges and struggled further with the complexities and frequency of required weekly lab tests and pharmacy visits, especially in rural areas. Finally, the implication of “needing” a REMS increased the perception of risk by patients and providers, which led to many only considering clozapine as a final resort.

While not as well‐covered within movement disorder literature, the consequences of the REMS program have been thoroughly described in the schizophrenia literature. Although roughly one‐quarter of individuals with schizophrenia fail to respond adequately to non‐clozapine antipsychotics,^26^ the initiation of clozapine is frequently delayed by years in countries with significant regulatory or procedural barriers such as the REMS,^27^ and many patients never receive an adequate trial at all. This has significant consequences—postponing clozapine initiation and enduring multiple unsuccessful antipsychotic trials markedly diminishes the medication's ultimate effectiveness in schizophrenia.^27^ Although PD and DLB have not been specifically included in studies documenting reduced clozapine efficacy with delayed initiation, there is little doubt that uncontrolled psychosis carries significant risks of morbidity and mortality. Opting for a less effective treatment solely because it is more convenient or easier to manage may come at substantial cost to patient outcomes.^3^

## The End of the REMS Program and Clinical Implications

Recently, these logistical barriers associated with prescribing clozapine in the US were dramatically reduced. Through persistent advocacy from patients, families, pharmacists, psychiatrists, and other invested parties, the FDA announced in late February that it would no longer require compliance with the REMS program and directed manufacturers of clozapine to discontinue the program.^28^ This came after a thorough re‐review of the risks surrounding the medication and finding that the data support that there is no need for a REMS. While the risk of serious side effects to clozapine, including severe neutropenia and myocarditis, appear dose‐ independent,^29^ the risk decreases significantly over a longer treatment duration, with the highest risk in the first month of exposure and a decrease to minimal levels after a year.^30^ Adding to this, in a recent large cohort, there was no increased risk in monitoring every three months rather than every month after one year of treatment.^31^

Although the FDA recommended continuing the previously established blood monitoring guidelines, the change acknowledged clozapine's unique efficacy in treating psychosis and the importance of individualizing the assessment of risks and benefits to each patient. Without the existence of the REMS, decisions regarding monitoring are now at the level of the patient and provider rather than dictated by institutional oversight. This has major, disease‐specific implications with many patients who could benefit from expanded clozapine usage.

### Psychosis in Parkinson's Disease and Dementia with Lewy Bodies

In DLB, psychosis often develops earlier, and thus patients could potentially be on clozapine for a longer period of time. It may be helpful to counsel patients on the limited benefit of continued blood testing after one year, which could impact medication adherence when patients, families, and providers could consider this a time‐limited barrier with a clear end date.

Conversely, psychosis in PD tends to occur later in the course of disease, and management of psychosis often occurs in the context of larger palliative care discussions. Given the low incidence of severe neutropenia and the high patient and caregiver morbidity associated with psychosis or agitation, it may even be reasonable to forego lab testing entirely or substantially truncate the period in which this is done.

Nonetheless, providers should always discuss clozapine's risk of severe neutropenia and other side effects with patients and families, as well as alternatives and the risk of no treatment, and clinical decision‐making must remain individualized, weighing potential risks and benefits based on patient‐specific factors, as is standard in medical practice. It is also worth noting in addition to the neutropenia risk, there is a black‐box warning for increased mortality with all antipsychotic medications (including clozapine, pimavanserin and quetiapine) in elderly patients with dementia^32^ that must be thoroughly discussed with patients and caregivers when considering initiation. However, psychosis is common and is one of the most distressing and functionally devastating aspects of these diseases,^33^ contributing to agitation, falls, institutionalization, and reduced survival^34^, ^35^—ultimately impacting both the quantity and quality of life.

### Other Applications of Clozapine

There are several other patient populations for whom clozapine has demonstrated efficacy and for whom the REMS represented a barrier to treatment. Tardive dyskinesia (TD) and other tardive movements that are related to long‐term antipsychotic use have been shown to improve or remit with a switch from the primary antipsychotic to clozapine.^36^ Clozapine has some case‐series level evidence for dystonia, and given the limited pharmacological options could be considered in severe cases.^37^ In Huntington's disease (HD), clozapine has mixed evidence for movement symptoms, with one double‐blind, randomized, placebo‐controlled trial showing reduction of chorea in antipsychotic‐naïve patients but with a high rate of adverse events.^38^ It is also used for severe psychiatric symptoms of HD, also with mixed efficacy.^39^ Finally, clozapine has evidence for Parkinsonian tremor (including tremor refractory to other therapies), with 58.5% reporting improvement in a large retrospective analysis.^6^ As with psychosis, it is likely underutilized in this patient population.^3^

## Conclusion

While the FDA's removal of the REMS has been widely discussed within psychiatry, much of this discourse has centered on schizophrenia rather than the unique implications for patients with Movement Disorders. Despite the potentially significant impact on patients with PD, DLB, and others, and especially the providers who care for them, advocacy within this field has been notably limited. In Fall of 2024, an FDA advisory committee convened to review the REMS program and voted 14‐1 in favor of abolishing the program, effectively paving the way for the FDA to do so earlier in 2025. Notably, among the hundreds of advocacy letters supporting the deregulation of clozapine prescribing, not a single neurological organization was represented. We feel that this is reflective of the longstanding underuse of this powerful weapon in the neuropsychiatric armamentarium, and hope that with the removal of the REMS this will remove a major logistical barrier to prescribing, ultimately leading to more effective treatment of PD and DLB psychosis.

## Author Roles

(1) Research: A. Conception, B. Organization, C. Execution; (2) Manuscript Preparation: A. Writing of the first draft, B. Review and Critique.

C.P.: 1A, 1B, 1C, 2A, 2B

A.M.: 1A, 1B, 1C, 2A, 2B

F.R.P.: 1A, 1B, 1C, 2B

## Disclosures

**Funding Sources and Conflicts of Interest:** No specific funding was received for this work. The authors declare that there are no conflicts of interest relevant to this work.

**Financial Disclosures for the Previous 12 Months:** The authors declare that there are no additional disclosures to report.

**Ethical Compliance Statement:** The authors confirm that the approval of an institutional review board was not required for this work, nor was informed consent. We confirm that we have read the Journal's position on issues involved in ethical publication and affirm that this work is consistent with those guidelines.

## Supporting information


**Supplemental Table S1.** Summary of studies evaluating clozapine on Parkinson's Disease psychosis. Studies are listed chronologically with reported study characteristics. When available, specific scale results are listed, otherwise results related to “clinical report” include any improvement, including complete resolution, significant improvement, moderate improvement, and mild or partial improvement. Reported cases of leukopenia and neutropenia and outcomes (such as transient or leading to discontinuation) are listed. BPRS, Brief Psychiatric Rating Scale; CBD, corticobasal degeneration; CGI, Clinical Global Impression Scale; CGI‐C, Clinical Global Impression of Change Scale; DLB, Dementia with Lewy Bodies; NPI, Neuropsychiatric Inventory; PDP, Parkinson's Disease with psychosis; PPRS, Parkinsonian Psychotic Rating Scale; PRS, Psychiatric Rating Scale.

## Data Availability

Data sharing is not applicable to this article as no new data were created or analyzed in this study.
