# Supplementary material for: Discontinuation of the Clozapine Risk Evaluation and Mitigation Strategy and Implications in Movement Disorders
Source: Mov Disord Clin Pract. 2025 Jun 28;12(12):2106–10. doi: 10.1002/mdc3.70197 (PMC12715328; doi:10.1002/mdc3.70197)
Supplement: Supplementary file 1 — Supplemental Table S1. Summary of studies evaluating clozapine on Parkinson's Disease psychosis. Studies are listed chronologically with reported study characteristics. When available, specific scale results are listed, otherwise results related to “clinical report” include any improvement, including complete resolution, significant improvement, moderate improvement, and mild or partial improvement. Reported cases of leukopenia and neutropenia and outcomes (such as transient or leading to discontinuation) are listed. BPRS, Brief Psychiatric Rating Scale; CBD, corticobasal degeneration; CGI, Clinical Global Impression Scale; CGI‐C, Clinical Global Impression of Change Scale; DLB, Dementia with Lewy Bodies; NPI, Neuropsychiatric Inventory; PDP, Parkinson's Disease with psychosis; PPRS, Parkinsonian Psychotic Rating Scale; PRS, Psychiatric Rating Scale. [file MDC3-12-2106-s001.docx]

Supplemental Table 1. Summary of studies using clozapine for Parkinson’s Disease psychosis.

| Study | Design | Population | N | Average Treatment Duration | Average Dose | Psychosis Outcome | Improvement | Leukopenia and Neutropenia Cases |
| --- | --- | --- | --- | --- | --- | --- | --- | --- |
| Kahn, 1991^1^ | Open Label | PDP | 11 | 8 months | 56mg | Clinical report | 72% | 0/0 |
| Factor, 1994^2^ | Open label | PDP | 17 | 15 months | 49mg | PRS | 49% | 0/0 |
| Rabey, 1995^3^ | Open label | PDP | 27 | 6.8 months | 42mg | Clinical report | 92% | 0/0 |
| Wagner, 1996^4^ | Chart Review | PDP | 49 | 18 months | 31mg | Clinical report | 76% | 2 (transient)/0 |
| Ruggieri, 1997^5^ | Open label | PDP | 36 | 12 months | 10.59mg | BPRS | Reduction from 20 to 8 | 0/0 |
| Trosch et al 1998^6^ | Chart Review | PDP | 172 | 16.7 months | 31.4mg | Clinical report | 89.5%-90.6% | 0/4 (transient) |
| PSG, 1999^7^ | RCT (placebo controlled) | PDP | 54 | 28 days | 24.7mg | CGI | -1.5 compared with placebo -0.5 | 0/0 |
| Klein, 2003^8^ | Chart Review | PDP | 32 | 6-60 months | 20mg | PPRS | 100% | 0/0 |
| Pollak, 2004^9^ | RCT (placebo controlled) | PDP | 60 | 28 days | 35.8mg | CGI | -1.8 compared to placebo 0.8 | 0/0 |
| Fernandez, 2004^10^ | Chart Review | PDP, DLB | 39 | >24 months | 47mg | Clinical report | 98% | 2 (transient)/0 |
| Merims, 2006^11^ | RCT (quetiapine comparison) | PDP | 14 | 22 weeks | 13.1mg | NPI and CGI-C | NPI: Hallucinations, 3 to 1.5; Delusions 3 to 1  CGI-C: 2.3 to 1 | 1/1 (significant) |
| Gomide, 2008^12^ | Chart Review | PDP and/or dyskinesia | 43 | 17 patients <1 year, 26 patients mean 3.5 years | 69.6mg | Clinical report | 73% | 2/3 (led to discontinuation) |
| Thomas, 2010^13^ | Chart Review | PDP, DLB, CBD and Benedikt syndrome | 53 | 39.9 months | 64.7mg | Clinical report | 77% | 2 (transient)/0 |
| Hack 2014^14^ | Chart Review | PDP | 32 | Not listed | Not listed | Clinical report | 71.9% | 0/3 (transient) |
| Friedman 2022^15^ | Chart Review | PDP, tremor/dyskinesia, DLB | 65 | Variable | 40.1mg | Clinical report | 75% | 0/1 (agranulocytosis) |
| Thames, 2023^16^ | Open label | Pimavans-erin refractory PDP | 27 | 17 months | 49.5mg | Clinical report | 63% | 0/0 |
| Pirker, 2025^17^ | Chart Review | Quetiapine refractory PDP | 38 | 30.6 months | 72.9mg | Clinical report | 94% | 0/1 (agranulocytosis) |

Table 1. Summary of studies evaluating clozapine on Parkinson’s Disease psychosis. Studies are listed chronologically with reported study characteristics. When available, specific scale results are listed, otherwise results related to “clinical report” include any improvement, including complete resolution, significant improvement, moderate improvement, and mild or partial improvement. Reported cases of leukopenia and neutropenia and outcomes (such as transient or leading to discontinuation) are listed.

BPRS: Brief Psychiatric Rating Scale; CBD: corticobasal degeneration; CGI: Clinical Global Impression Scale; CGI-C: Clinical Global Impression of Change Scale; DLB: Dementia with Lewy Bodies; NPI: Neuropsychiatric Inventory; PDP: Parkinson’s Disease with psychosis; PPRS: Parkinsonian Psychotic Rating Scale; PRS: Psychiatric Rating Scale

1. Kahn N, Freeman A, Juncos JL, et al. Clozapine is beneficial for psychosis in Parkinson's disease. *Neurology* 1991;41(10):1699-99. doi: doi:10.1212/WNL.41.10.1699

2. Factor SA, Brown D, Molho ES, et al. Clozapine: a 2-year open trial in Parkinson's disease patients with psychosis. *Neurology* 1994;44(3 Pt 1):544-6. doi: 10.1212/wnl.44.3_part_1.544

3. Rabey JM, Treves TA, Neufeld MY, et al. Low-dose Clozapine in the Treatment of Levodopa-Induced Mental Disturbances in Parkinson's Disease. *Neurology* 1995;45(3):432-34. doi: doi:10.1212/WNL.45.3.432

4. Wagner ML, Defilippi JL, Menza MA, et al. Clozapine for the treatment of psychosis in Parkinson's disease: chart review of 49 patients. *J Neuropsychiatry Clin Neurosci* 1996;8(3):276-80. doi: 10.1176/jnp.8.3.276

5. Ruggieri S, De Pandis MF, Bonamartini A, et al. Low dose of clozapine in the treatment of dopaminergic psychosis in Parkinson's disease. *Clin Neuropharmacol* 1997;20(3):204-9. doi: 10.1097/00002826-199706000-00003

6. Trosch RM, Friedman JH, Lannon MC, et al. Clozapine use in Parkinson's disease: a retrospective analysis of a large multicentered clinical experience. *Mov Disord* 1998;13(3):377-82. doi: 10.1002/mds.870130302

7. Group TPS. Low-dose clozapine for the treatment of drug-induced psychosis in Parkinson's disease. *The New England journal of medicine* 1999;340(10):757-63. doi: 10.1056/nejm199903113401003

8. Klein C, Gordon J, Pollak L, et al. Clozapine in Parkinson's disease psychosis: 5-year follow-up review. *Clin Neuropharmacol* 2003;26(1):8-11. doi: 10.1097/00002826-200301000-00003

9. Pollak P, Tison F, Rascol O, et al. Clozapine in drug induced psychosis in Parkinson’s disease: a randomised, placebo controlled study with open follow up. *Journal of Neurology, Neurosurgery &amp; Psychiatry* 2004;75(5):689-95. doi: 10.1136/jnnp.2003.029868

10. Fernandez HH, Donnelly EM, Friedman JH. Long-term outcome of clozapine use for psychosis in parkinsonian patients. *Mov Disord* 2004;19(7):831-33. doi: 10.1002/mds.20051

11. Merims D, Balas M, Peretz C, et al. Rater-blinded, prospective comparison: quetiapine versus clozapine for Parkinson's disease psychosis. *Clin Neuropharmacol* 2006;29(6):331-7. doi: 10.1097/01.Wnf.0000236769.31279.19

12. Gomide L, Kummer A, Cardoso F, et al. Use of clozapine in Brazilian patients with Parkinson's disease. *Arq Neuropsiquiatr* 2008;66(3b):611-4. doi: 10.1590/s0004-282x2008000500001

13. Thomas AA, Friedman JH. Current use of clozapine in Parkinson disease and related disorders. *Clin Neuropharmacol* 2010;33(1):14-6. doi: 10.1097/WNF.0b013e3181c47168

14. Hack N, Fayad SM, Monari EH, et al. An eight-year clinic experience with clozapine use in a Parkinson's disease clinic setting. *PLoS One* 2014;9(3):e91545. doi: 10.1371/journal.pone.0091545 [published Online First: 20140319]

15. Friedman JH, Hershkowitz D. Clozapine Use in a Movement Disorder Clinic. *Clinical Neuropharmacology* 2022;45(4):95-98. doi: 10.1097/wnf.0000000000000510

16. Thames BH, Ondo WG. Clozapine: Efficacy for Parkinson Disease psychosis in patients refractory to pimavanserin. *Parkinsonism Relat Disord* 2023;109:105356. doi: 10.1016/j.parkreldis.2023.105356 [published Online First: 20230305]

17. Pirker W. Clozapine for Quetiapine-Refractory Psychosis in Parkinson's Disease: A Long-Term Single-Center Retrospective Study. *Parkinsons Dis* 2025;2025:1068722. doi: 10.1155/padi/1068722 [published Online First: 20250310]
